# Vomiting in Pediatric Patients

**DOI:** 10.21980/J8P363

**Published:** 2020-10-15

**Authors:** Alisa Wray, Daryn Towle, Alexa Lucas, Sean Thompson, Katie Rebillot, Nichole Niknafs

**Affiliations:** * University of California, Irvine, Department of Emergency Medicine, Orange, CA; ^ Los Angeles County-Keck School of Medicine, University of Southern California, Department of Emergency Medicine, Los Angeles, CA; † Arrowhead Regional Medical Center, Department of Emergency Medicine, Colton, CA

## Abstract

**Audience:**

This classic team-based learning activity is specifically designed for emergency medicine bound medical students and junior residents; however, general pediatrics residents and general medical students may also benefit from this activity. Senior residents and fellows felt that the cases were too basic for them but enjoyed acting as facilitators.

**Introduction/Background:**

Vomiting is a common chief complaint in pediatric patients seen in the Emergency Department. 1–3 Presentations include acute, chronic, and cyclic vomiting, with underlying etiologies such as toxin injection, emotional disturbances, and movement disequilibrium. 1 By understanding these various pathways, it is helpful for physicians to distinguish between gastrointestinal and non-gastrointestinal causes of vomiting. 1 Most cases of vomiting in the pediatric population are self-limiting and require only supportive treatment; however, physicians must be able to recognize red flags associated with vomiting that warrant further evaluation. 1,3 This task may be challenging for medical students and residents in emergency medicine and those with infrequent exposure to pediatric patients. Therefore, this team-based learning activity was developed to help junior learners in differentiating non-emergent and emergent cases of pediatric vomiting. This activity aids learners in formulating a differential based on age, history, and characteristics of vomiting. We also review specific causes of pediatric vomiting that physicians cannot miss including intussusception, pyloric stenosis, malrotation, intestinal atresia, and intracranial pathology.

**Educational Objectives:**

By the end of this TBL session, learners should be able to:

1. Identify red flag symptoms that should prompt referral for urgent intervention by GI or surgical specialists.
2. Recognize how chronicity of the vomiting can alter the differential diagnosis
3. Describe the varying pathways that can cause nausea and vomiting.
4. Determine the necessity of imaging tests to confirm and possibly treat various causes of vomiting.
5. Interpret imaging studies associated with specific causes of vomiting.

**Educational Methods:**

Classic Team Based Learning (cTBL)

**Research Methods:**

Learners and instructors provided verbal feedback after the session in a large group format. Learners were specifically asked if they felt the session was education, relevant, high-yield and level appropriate. One instructor provided written feedback to the cases as well.

**Results:**

Overall learners and instructors found the session to be engaging, informative and educational. Learners felt that the session was level appropriate for medical students and junior residents. As a result of feedback from the session, several of the iRAT/gRAT questions were adjusted and the group application cases were re-written and implemented.

**Discussion:**

Overall, the educational content and delivery was effective. This session was presented to a group of emergency medicine students, interns and residents. Learners were divided into smaller groups, and each group had a variety of level of learners, including pediatric emergency medicine fellows, present. The fellows, while not necessary to the delivery of the TBL, were extremely helpful in aiding the residents during the session. The final debriefing and answer review were essential to ensure that learners met all educational objectives and fully understood the materials.

**Topics:**

Pediatric vomiting, intussusception, pyloric stenosis, intestinal atresia, malrotation, gastroesophageal reflux disease, superior mesenteric artery (SMA) syndrome, hyperemesis.

## USER GUIDE

**Table:** 

List of Resources:	
Abstract	1
User Guide	3
Learner Materials	6
iRAT	6
gRAT	11
GAE	17
Instructor Materials	25
RAT Key	26
GAE Key	34

**Learner Audience:**

Medical students, interns, junior residents

**Time Required for Implementation:**

Instructor Preparation: 60 minutes

Learner Responsible Content: 15–30 minutes

In Class Time: 90 minutes

**Recommended Number of Learners per Instructor:**

This activity can include anywhere from 10 learners to 30 learners. Only one instructor is required during this activity. For larger groups with more than 30 learners, it may be helpful to have more instructors to facilitate discussion. For example, we utilized pediatric emergency medicine fellows as additional facilitators; additionally, senior residents could be used as facilitators.

**Topics:**

Pediatric vomiting, intussusception, pyloric stenosis, intestinal atresia, malrotation, gastroesophageal reflux disease, superior mesenteric artery (SMA) syndrome, hyperemesis.

**Objectives:**

By the end of this TBL session, learners should be able to:

1. Identify red flag symptoms that should prompt referral for urgent intervention by GI or surgical specialists.
2. Recognize how chronicity of the vomiting can alter the differential diagnosis
3. Describe the varying pathways that can cause nausea and vomiting.
4. Determine the necessity of imaging tests to confirm and possibly treat various causes of vomiting.
5. Interpret imaging studies associated with specific causes of vomiting.

### Linked objectives and methods

Pediatric vomiting is a frequent chief complaint encountered in the emergency department. The National Hospital Ambulatory Medical Care Survey (NHAMCS) showed 9.46% of pediatric emergency visits presented with vomiting as the chief complaint.^2^ There are a wide variety of differential diagnoses ranging from benign to critical which can present as vomiting. The majority of these children with vomiting will ultimately have uneventful courses and benign diagnoses; however, it is important for physicians to be aware of the critical diagnoses.^3^ Therefore, this team-based learning activity was designed to help learners differentiate between these causes of pediatric vomiting. The iRAT and gRAT questions broadly cover all of the objectives. Case 1 covers acute vomiting in a teenage female with a history of diabetic ketoacidosis (DKA), and it reviews pertinent blood tests required for acute vomiting and reminds learners to obtain a urine pregnancy test. Overall, Case 1 demonstrates objectives 2, 3, and 4 in the work up for the patient and discussion of the case questions. Case 2 covers and reviews the variety of reasons for vomiting in the neonate, objectives 1, 2, 3 and 4, by prompting learners to discuss and consider the differential diagnosis, blood work, and imaging that is necessary for an acutely vomiting neonate. Case 3 covers abdominal pain, vomiting, and GI bleeding in a younger pediatric patient with a final diagnosis of intussusception. Learners are asked to determine the labs and imaging they would like to order, prompting learning of objectives 1, 2, 3, 4 and 5. Lastly, case 4 covers the very important non-gastrointestinal causes of pediatric vomiting and prompts learners to perform full review of systems and physical exam, important for objectives 2 and 3. Throughout the activity, learners are encouraged to discuss the cases with each other and instructors.

### Recommended pre-reading for instructor

This TBL is based on the article: “Vomiting in Children” by T. Matthew Shields, MD, and Jenifer R. Lightdale, MD, MPH. We recommend that instructors read this article prior to administering the TBL so that they are prepared for the session. The article is available here: https://pedsinreview.aappublications.org/content/39/7/342. Alternatively, any article or book chapter on vomiting in pediatric patients would be appropriate. Please see the references for further suggestions.

### Learner Responsible Content (LRC)

This TBL is based on the article: “Vomiting in Children” by T. Matthew Shields, MD, and Jenifer R. Lightdale, MD, MPH. We recommend that learners read this article prior to the TBL so that they are prepared for the session. The article is available here: https://pedsinreview.aappublications.org/content/39/7/342.

### Results and tips for successful implementation

This Pediatric Vomiting TBL was tested on a group of 17 emergency residents including interns, junior and senior residents who completed the TBL, 5 pediatric emergency medicine fellows, and 4 medical students. The fellows acted as small groups facilitators and helped guide the discussions. Learners provided verbal feedback which was overall very positive. Residents commented that the session was “relevant,” “interactive,” and “high-yield”. Some senior level residents felt that the questions and cases were slightly too easy for them but still found them to be educational and good review. Pediatric emergency medicine fellows acted as faculty facilitators and reported that they enjoyed their role and felt that they were able to add to the discussion among residents and students. The pediatric emergency medicine fellows also provided verbal and written feedback on the cases, and several small changes to the iRAT/gRAT and GAE cases were suggested, and edits to the final TBL were made.

Prior to session, instructor will need to prepare:

- One iRAT (individual readiness assessment test) per learner
- One gRAT (group readiness assessment test) per group (ideally 3–5 learners per group)
  - Use scratch off stickers https://www.amazon.com/Kenco-Scratch-Off-Stickers-Silver/dp/B0839QF79D/ref=sr_1_8?dchild=1&keywords=scratch+off+stickers&qid=1586830370&sr=8-8 to hide the answers/stars so that learners get immediate feedback on their answer choice. See the example below.
    **Figure:**
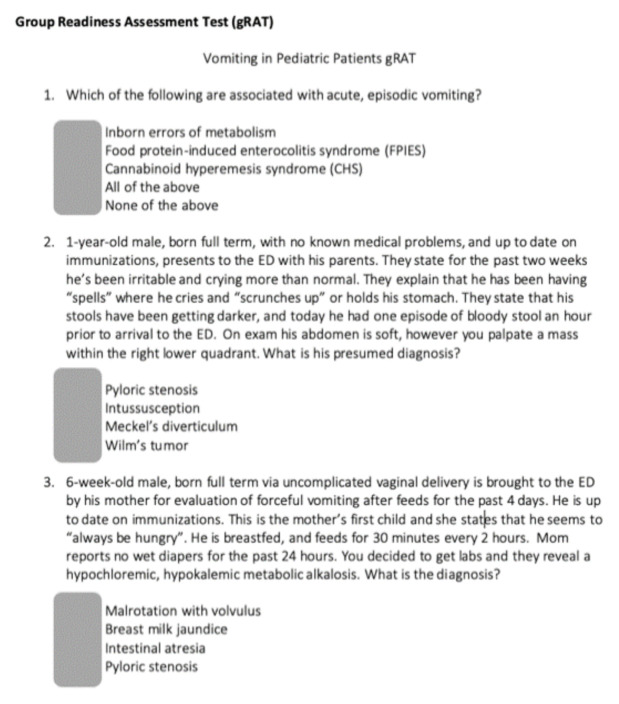
- One group application exercise per group
  We used post-it notes to hide the labs and images in the cases so that learners could remove the post-it and see the images after they had committed to their orders for the patients. See example below.
    **Figure:**
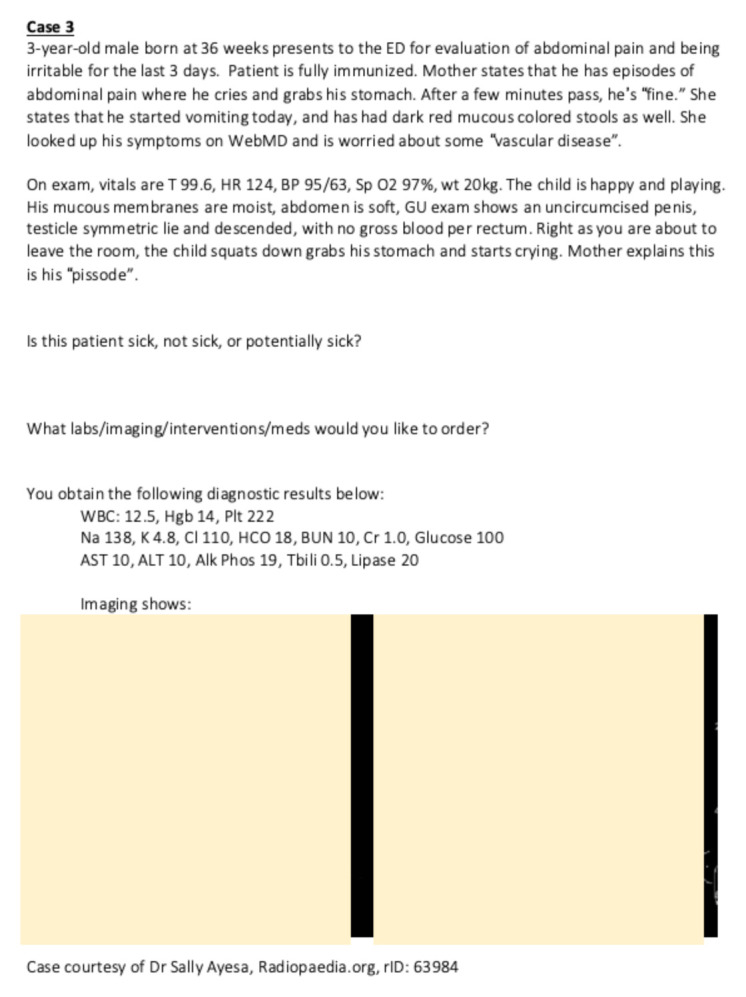

Conducting the session:

- Introduce the topic; if desired provide learners with the article “Vomiting in Children” by T. Matthew Shields, MD, and Jenifer R. Lightdale, MD, MPH, prior to the session or at the beginning of the session for learners to read prior to the iRAT.
- Instructor disperses iRAT to all learners. These should be completed individually (5–10 minutes).
- Instructor then assigns groups of 3–5 learners and disperses gRAT to all groups to complete (5–10 minutes). When assigning our groups, we assigned a variety of learners, medical students and junior residents with 1 to 2 senior residents or fellows to help facilitate.
- Instructor then reviews the readiness assessment test and answers any questions individuals may have related to the test (5–10 minutes).
- Instructor then hands out the group application exercise. Groups should complete all cases. (30 minutes).
- Answers to GAE are reviewed. It is recommended to have each group answer one case and explain their thought process to provide for discussion from all learners (30 minutes).

## Figures and Tables

**Figure:**
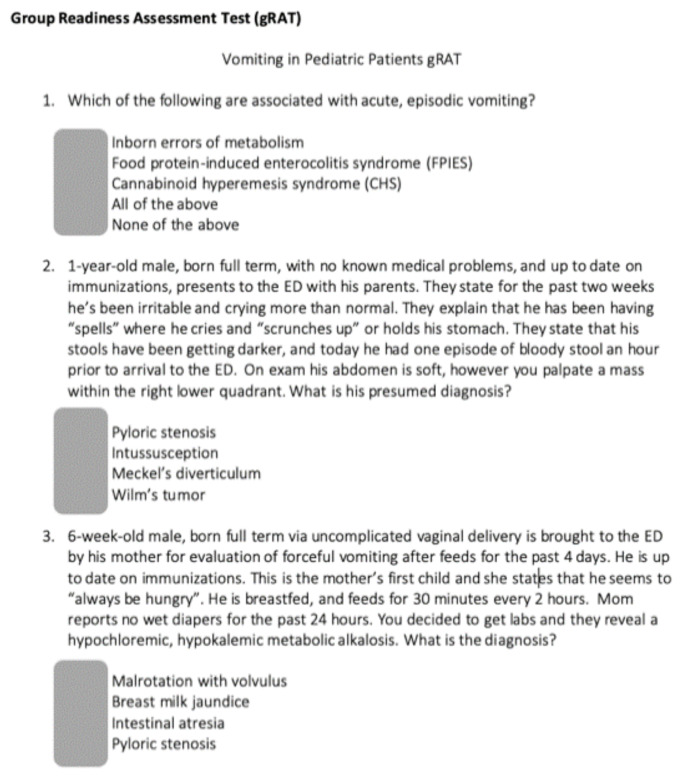


**Figure:**
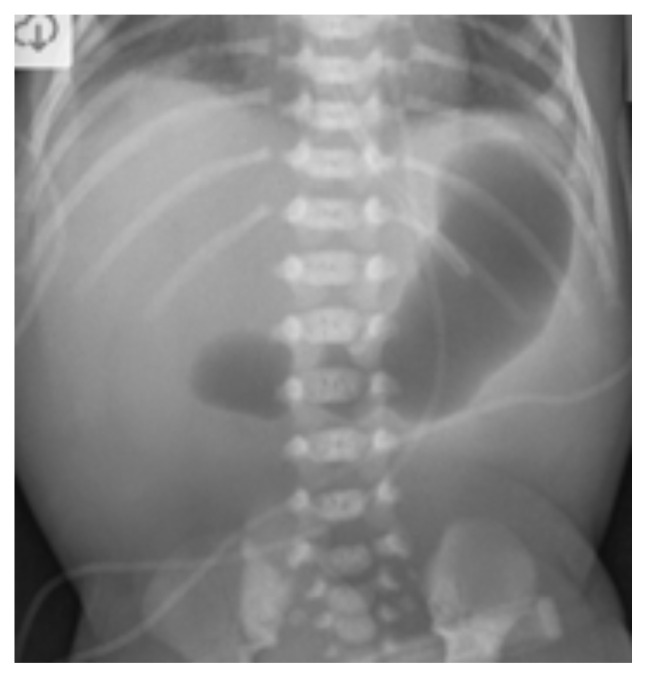


**Figure:**
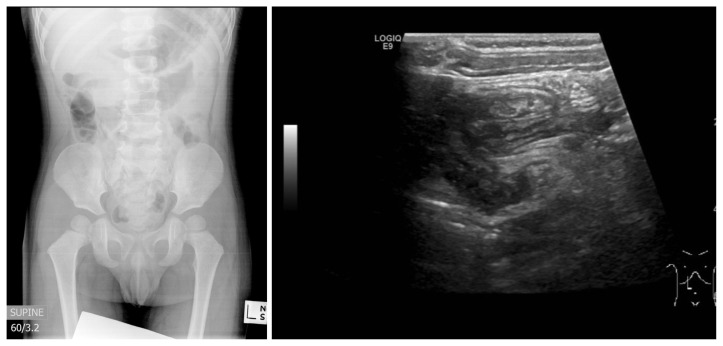


**Figure:**
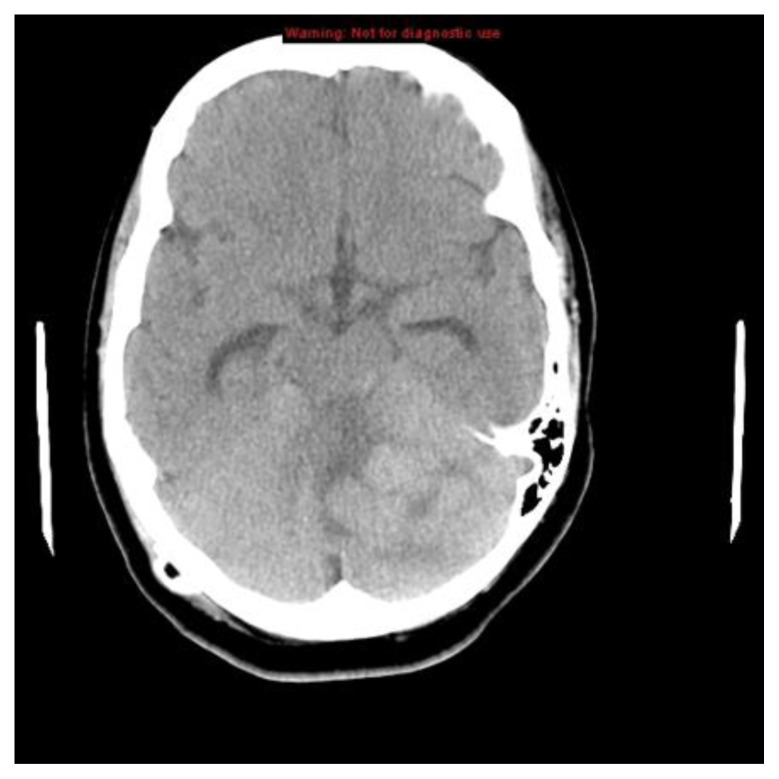


**Figure:**
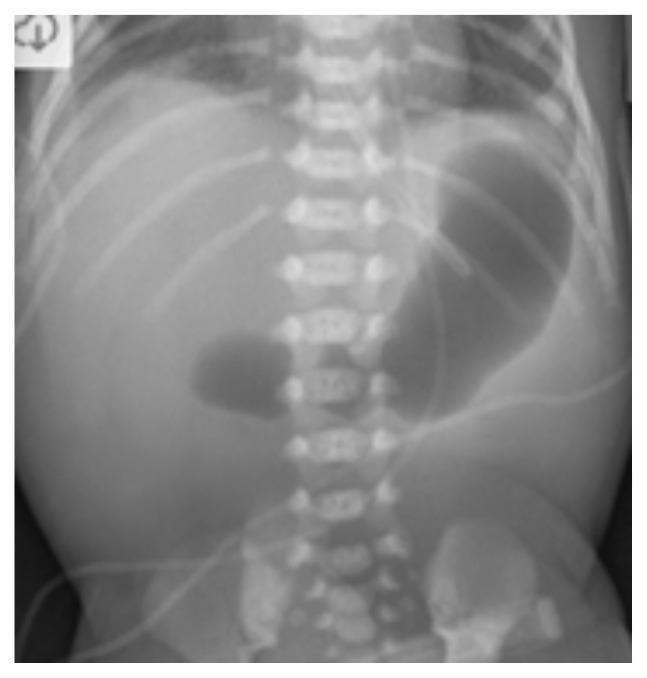


**Figure:**
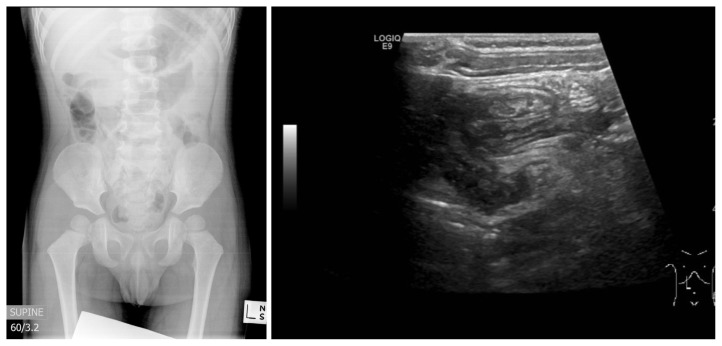


**Figure:**
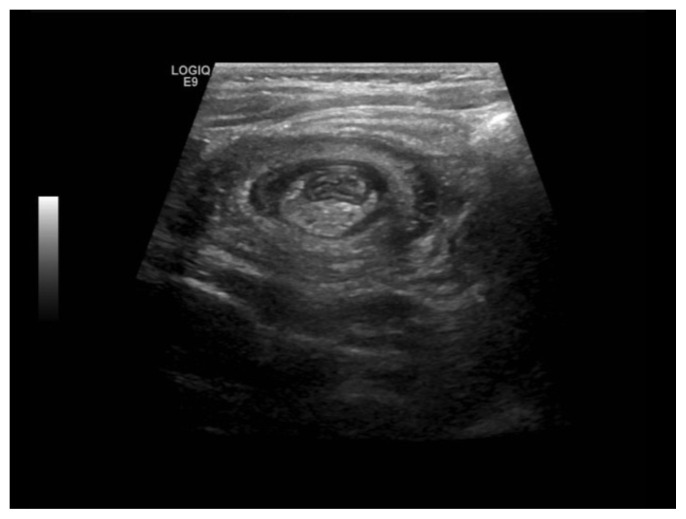


**Figure:**
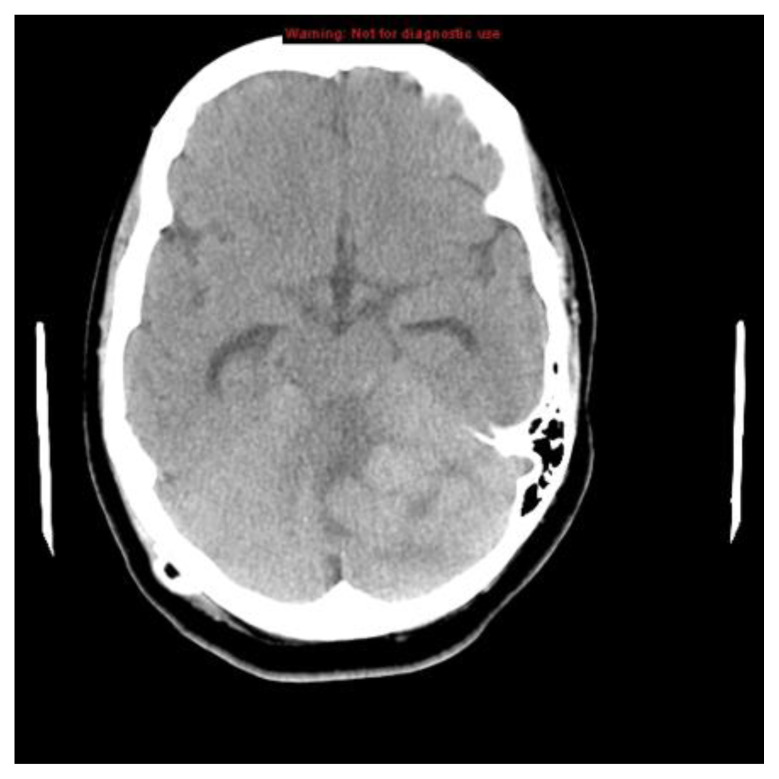


## LEARNER MATERIALS

### Vomiting in Pediatric Patients TBL: individual Readiness Assessment Test (iRAT)

1. Which of the following are associated with acute, episodic vomiting?
  - Inborn errors of metabolism
  - Food protein-induced enterocolitis syndrome (FPIES)
  - Cannabinoid hyperemesis syndrome (CHS)
  - All of the above
  - None of the above
2. 1-year-old male, born full term, with no known medical problems, and up to date on immunizations, presents to the ED with his parents. They state for the past two weeks he’s been irritable and crying more than normal. They explain that he has been having “spells” where he cries and “scrunches up” or holds his stomach. They state that his stools have been getting darker, and today he had one episode of bloody stool an hour prior to arrival to the ED. On exam his abdomen is soft; however, you palpate a mass within the right lower quadrant. What is his presumed diagnosis?
  - Pyloric stenosis
  - Intussusception
  - Meckel’s diverticulum
  - Wilm’s tumor
3. 6-week-old male, born full term via uncomplicated vaginal delivery is brought to the ED by his mother for evaluation of forceful vomiting after feeds for the past 4 days. He is up to date on immunizations. This is the mother’s first child, and she states that he seems to “always be hungry.” He is breastfed, and feeds for 30 minutes every 2 hours. Mom reports no wet diapers for the past 24 hours. You decided to get labs and they reveal a hypochloremic, hypokalemic metabolic alkalosis. What is the diagnosis?
  - Malrotation with volvulus
  - Breast milk jaundice
  - Intestinal atresia
  - Pyloric stenosis
4. A 24-month-old male is brought in to the ED with by his dad for vomiting for the last 24 hours. The dad reports that the whole family has been ill since attending a lunch picnic yesterday. On physical exam the patient is mildly tachycardic, has dry mucous membranes, and a soft minimally tender abdomen. The first step in management is:
  - Abdominal radiograph
  - Antiemetics and fluids
  - Antibiotics for likely bacterial colitis
  - NG tube for bowel decompression
5. 7-month-old female, born full term and up to date on immunizations presents for evaluation of bilious emesis; parents deny bloody stools. On exam, the child is sick appearing and abdomen is distended. You order a stat KUB (kidneys, ureters, bladder) which shows air-filled levels, and dilated stomach and bowel in the upper quadrants with a paucity of air distally.
  What is your diagnosis and treatment?
  - Malrotation with volvulus; decompression with nasogastric intubation (NGT) and surgery consult
  - Pyloric stenosis; pyloromyotomy
  - Intestinal atresia; surgery consult
  - Intussusception; air enema
6. Which of the following are “Red Flag” signs & symptoms that require urgent evaluation?
  - Hematemesis/Hematochezia
  - Bilious emesis
  - Abdominal distension/absent bowel sounds
  - Vomiting that wakes child from sleep
  - All the above
7. 4-month-old female, born term and up to date on immunizations, presents to the ED for evaluation of being more irritable and fussier after feeds. She’s breastfed solely. Mother states that she has had issues with “spitting up” after feeds but has always been “happy,” and even though she appears hungry mom feels like the patient is losing weight. What is the most likely diagnosis?
  - GERD (gastroesophageal reflux disease)
  - Breastfeeding jaundice
  - Milk protein allergy
  - Eosinophilic esophagitis
8. 3-year-old boy presents with colicky abdominal pain and non-bilious vomiting. He appears non-toxic. Parents state during these episodes he cries uncontrollably and at times they wake him up from sleep for the past week. They deny bloody stools. What is the diagnostic study to best evaluate and treat this patient?
  - Ultrasound
  - Air contrast enema
  - Surgery
  - EGD (esophagogastroduodenoscopy)
9. You are on your NICU rotation and called to come evaluate a newborn male with history of trisomy 21. The baby has been having bilious vomiting after his first feed with a distended abdomen. You order a stat KUB which shows a large gastric bubble and then a distal smaller bubble of air. What is your presumed diagnosis?
  - Intestinal atresia
  - Pyloric Stenosis
  - NEC (necrotizing enterocolitis)
  - GERD
10. An 8-year-old boy presents with acute vomiting that began after lunch yesterday with one to two episodes, but then became intractable late last night. The patient has dry mucous membranes, his abdomen is soft, non-tender and non-distended. His vital signs are HR 120, BP 90/84, RR 18. What diagnostic studies would you order?
  - Chem 7 & KUB
  - Chem 7, LFTs (liver function tests), amylase/lipase, EGD, MRI/CT head
  - Chem 7, ammonia, pyruvate, abdominal US, urine studies
  - Urine studies only
11. The pathways of emetic reflex are best described by:
  - Motion
  - Emotional Responses
  - Toxins introduced by drugs/food/environment
  - Eating too much
  - All the above
12. 14-year-old female with history of migraines presents to the ER for evaluation of vomiting spells for the past week that occur upon waking up from sleep. She’s had an MRI and CT head that have been negative for masses. The patient’s mother states that with her migraines she only gets headaches, and never experiences nausea or vomiting. Which elements of the history are important to obtain in order to help you with your diagnosis?
  - Social
  - Alleviating factors
  - Sexual & Surgical
  - Birth
  - All the above
13. A 16-year-old male presents to his primary care physician’s office with complaints of episodic post-prandial vomiting. He reports that it started after he lost 20 pounds when he joined the high school football team. He reports that when he eats he gets crampy abdominal pain, but it improves if he leans forward. What do you expect to see on upper GI series?
  - A sharp cutoff sign where the contrast is unable to pass through the duodenum
  - Distended small bowel with air fluid levels
  - Diffuse constipation
  - Dilation of the esophagus with no contrast past the lower esophageal sphincter

### Vomiting in Pediatric Patients TBL: group Readiness Assessment Test (gRAT)

Use scratch off stickers https://www.amazon.com/Kenco-Scratch-Off-Stickers-Silver/dp/B0839QF79D/ref=sr_1_8?dchild=1&keywords=scratch+off+stickers&qid=1586830370&sr=8-8 to hide the answers/stars so that learners get immediate feedback on their answer choice. See the example below.

### Vomiting in Pediatric Patients TBL: group Readiness Assessment Test (gRAT)

1. Which of the following are associated with acute, episodic vomiting?
  a. Inborn errors of metabolism
  b. Food protein-induced enterocolitis syndrome (FPIES)
  c. Cannabinoid hyperemesis syndrome (CHS)
  ★. All of the above
  e. None of the above
2. 1-year-old male, born full term, with no known medical problems, and up to date on immunizations, presents to the ED with his parents. They state for the past two weeks he’s been irritable and crying more than normal. They explain that he has been having “spells” where he cries and “scrunches up” or holds his stomach. They state that his stools have been getting darker, and today he had one episode of bloody stool an hour prior to arrival to the ED. On exam his abdomen is soft; however, you palpate a mass within the right lower quadrant. What is his presumed diagnosis?
  a. Pyloric stenosis
  ★. Intussusception
  c. Meckel’s diverticulum
  d. Wilm’s tumor
3. 6-week-old male, born full term via uncomplicated vaginal delivery is brought to the ED by his mother for evaluation of forceful vomiting after feeds for the past 4 days. He is up to date on immunizations. This is the mother’s first child, and she states that he seems to “always be hungry.” He is breastfed, and feeds for 30 minutes every 2 hours. Mom reports no wet diapers for the past 24 hours. You decided to get labs and they reveal a hypochloremic, hypokalemic metabolic alkalosis. What is the diagnosis?
  a. Malrotation with volvulus
  b. Breast milk jaundice
  c. Intestinal atresia
  ★. Pyloric stenosis
4. A 24-month-old male is brought in to the ED with by his dad for vomiting for the last 24 hours. The dad reports that the whole family has been ill since attending a lunch picnic yesterday. On physical exam the patient is mildly tachycardic, has dry mucous membranes, and a soft minimally tender abdomen. The first step in management is:
  a. Abdominal radiograph
  ★. Antiemetics and fluids
  c. Antibiotics for likely bacterial colitis
  d. NG tube for bowel decompression
5. 7-month-old female, born full term and up to date on immunizations presents for evaluation of bilious emesis; parents deny bloody stools. On exam, the child is sick appearing and abdomen is distended. You order a stat KUB (kidneys, ureters, bladder) which shows air-filled levels, and dilated stomach and bowel in the upper quadrants with a paucity of air distally.
  What is your diagnosis and treatment?
  ★. Malrotation with volvulus; decompression with nasogastric intubation (NGT) and surgery consult
  b. Pyloric stenosis; pyloromyotomy
  c. Intestinal atresia; surgery consult
  d. Intussusception; air enema
6. Which of the following are “Red Flag” signs & symptoms that require urgent evaluation?
  a. Hematemesis/Hematochezia
  b. Bilious emesis
  c. Abdominal distension/absent bowel sounds
  d. Vomiting that wakes child from sleep
  ★. All the above
7. 4-month-old female, born term and up to date on immunizations, presents to the ED for evaluation of being more irritable and fussier after feeds. She’s breastfed solely. Mother states that she has had issues with “spitting up” after feeds but has always been “happy,” and even though she appears hungry mom feels like the patient is losing weight. What is the most likely diagnosis?
  ★. GERD (gastroesophageal reflux disease)
  b. Breastfeeding jaundice
  c. Milk protein allergy
  d. Eosinophilic esophagitis
8. 3-year-old boy presents with colicky abdominal pain and non-bilious vomiting. He appears non-toxic. Parents state during these episodes he cries uncontrollably and at times they wake him up from sleep for the past week. They deny bloody stools. What is the diagnostic study to best evaluate and treat this patient?
  a. Ultrasound
  ★. Air contrast enema
  c. Surgery
  d. EGD (esophagogastroduodenoscopy)
9. You are on your NICU rotation and called to come evaluate a newborn male with history of trisomy 21. The baby has been having bilious vomiting after his first feed with a distended abdomen. You order a stat KUB which shows a large gastric bubble and then a distal smaller bubble of air. What is your presumed diagnosis?
  ★. Intestinal atresia
  b. Pyloric Stenosis
  c. NEC (necrotizing enterocolitis)
  d. GERD
10. An 8-year-old boy presents with acute vomiting that began after lunch yesterday with one to two episodes, but then became intractable late last night. The patient has dry mucous membranes, his abdomen is soft, non-tender and non-distended. His vital signs are HR 120, BP 90/84, RR 18. What diagnostic studies would you order?
  ★. Chem 7 & KUB
  b. Chem 7, LFTs (liver function tests), amylase/lipase, EGD, MRI/CT head
  c. Chem 7, ammonia, pyruvate, abdominal US, urine studies
  d. Urine studies only
11. The pathways of emetic reflex are best described by:
  a. Motion
  b. Emotional Responses
  c. Toxins introduced by drugs/food/environment
  d. Eating too much
  ★. All the above
12. 14-year-old female with history of migraines presents to the ER for evaluation of vomiting spells for the past week that occur upon waking up from sleep. She’s had an MRI and CT head that have been negative for masses. The patient’s mother states that with her migraines she only gets headaches, and never experiences nausea or vomiting. Which elements of the history are important to obtain in order to help you with your diagnosis?
  a. Social
  b. Alleviating factors
  c. Sexual & Surgical
  d. Birth
  ★. All the above
13. A 16-year-old male presents to his primary care physician’s office with complaints of episodic post-prandial vomiting. He reports that it started after he lost 20 pounds when he joined the high school football team. He reports that when he eats he gets crampy abdominal pain, but it improves if he leans forward. What do you expect to see on upper GI series?
  ★. A sharp cutoff sign where the contrast is unable to pass through the duodenum
  b. Distended small bowel with air fluid levels
  c. Diffuse constipation
  d. Dilation of the esophagus with no contrast past the lower esophageal sphincter

### Vomiting in Pediatric Patients TBL: Group Application Exercise (GAE)

#### Case 1

15-year-old female with history of Type 1 Diabetes presents with nausea and vomiting for the past week, accompanied by her mother. Patient states last menstrual period was 2 months ago, though she has irregular periods and denies being sexually active. She denies any bloody stools, abdominal pain, and has never been in DKA per mother.

On exam, vital signs are the following: T 98.6, RR 18, HR 100, BP 110/85. The patient appears pale and dry. Abdomen is soft though slightly distended, with normal bowel sounds. Pulmonary and cardiac exam are within normal limits.

Is this patient sick, not sick, or potentially sick?

What labs/interventions/meds would you like to order?

You obtain the following diagnostic results listed below. At this point what is your differential diagnosis?

- WBC: 10, Hbg 12, Plt 250
- Urinalysis: no leukesterase, no WBC, no RBC, no bacteria
- CMP: within normal limits
- Blood Glucose: 100

What other lab work would you like to order or did you order that has not resulted yet?

What imaging (if any) would you like to perform?

What treatment would you like to provide the patient?

#### Case 2

A 24-hour-old male born via C-Section at 33 weeks secondary to mother having pre-eclampsia presents to the ED. Quad screen was positive for trisomy 21. The labor and delivery nurse calls down and states that the baby was just discharged, and prior to discharge, they noted that he had an episode of bilious emesis and progressively worsening abdominal distention. The doctor called the family and asked them to return immediately. You note a birth weight 2400g, and the baby is formula fed. No bloody stools.

On exam, VS demonstrate T 98.6, HR 145/min, BP 91/60, and RR 50. The infant is lethargic-appearing with poor skin turgor. His abdomen is distended with normal bowel sounds. Pulmonary and cardiac exam are within normal limits.

Is this patient sick, not sick, or potentially sick?

What labs/interventions/meds would you like to order?

You obtain the following diagnostic results below:

WBC: 10, Hbg 11, Plt 253
Urine: within normal limits
CMP: within normal limits

At this point what is your differential diagnosis for this patient and what do you think is the most likely diagnosis?

You obtain the following radiograph:

Case courtesy of Dr Sebastian Tschauner, Radiopaedia.org, rID: 49592. https://radiopaedia.org/cases/49592

How would you describe the patient’s abdominal radiograph?

What is your diagnosis for this patient?

What is the treatment for this patient?

What is the prognosis for this patient?

#### Case 3

3-year-old male born at 36 weeks presents to the ED for evaluation of abdominal pain and being irritable for the last 3 days. Patient is fully immunized. Mother states that he has episodes of abdominal pain where he cries and grabs his stomach. After a few minutes pass, he’s “fine.” She states that he started vomiting today, and has had dark red mucous colored stools as well. She looked up his symptoms on WebMD and is worried about some “vascular disease.”

On exam, vitals are T 99.6, HR 124, BP 95/63, Sp O2 97%, wt 20kg. The child is happy and playing. His mucous membranes are moist, abdomen is soft, GU exam shows an uncircumcised penis, testicles symmetric and descended, with no gross blood per rectum. Right as you are about to leave the room, the child squats down grabs his stomach and starts crying. Mother explains this is his “episode.”

Is this patient sick, not sick, or potentially sick?

What labs/imaging/interventions/meds would you like to order?

You obtain the following diagnostic results below:

WBC: 12.5, Hgb 14, Plt 222
Na 138, K 4.8, Cl 110, HCO 18, BUN 10, Cr 1.0, Glucose 100
AST 10, ALT 10, Alk Phos 19, Tbili 0.5, Lipase 20

Imaging shows:

Case courtesy of Dr Sally Ayesa, Radiopaedia.org, rID: 63984. https://radiopaedia.org/cases/63984

What are common causes of lower GI bleeding found in kids in this age group?

What is the most likely diagnosis?

Bonus points: What are some pathological conditions associated with this condition?

What imaging would you use to confirm and ultimately treat the patient’s condition?

#### Case 4

A 16-year-old female presents for an intractable headache. She notes that the onset is first thing in the morning and that it wakes her from sleep and worsens throughout the day. Her mother states the symptoms have been progressively worsening and that the patient appears “off-balance” when she walks. Associated symptoms are nausea/vomiting; she denies blurred vision, hearing changes, weight loss, photophobia. She states her head hurts “all over.” She currently is on her menstrual period. She denies history of migraines. No pertinent family history. Social history is positive for occasional marijuana use.

On exam, vital signs are T 98.7, HR 98, RR 18, SpO2 97% RA. She is 60kg, and 5’7”. The patient is holding both sides of her head and crying; you perform a full neuro exam and notice she has drift on the right. The remainder of your exam is unremarkable.

Is this patient sick, not sick, or potential to be sick?

What labs/imaging/interventions/meds would you like to order?

You obtain the following results below:

WBC: 12, Hgb 13, Plt 235
CMP: within normal limits
UA: within normal limits
Urine pregnancy test: negative

What history elements are important to ask a pediatric patient with a headache?

What is the clinical significance of the associated symptom of vomiting that the patient is experiencing?

What is your differential diagnosis for this patient?

You obtain a computed tomography of the head:

Case courtesy of Dr Hani Salam, Radiopaedia.org, rID: 10374. https://radiopaedia.org/cases/10374

What is your next step in management of this patient?

## INSTRUCTOR MATERIALS

Answer keys to all exercises with explanations, are on the following pages.

Learners: Please do not proceed.

### Vomiting in Pediatric Patients TBL: Readiness Assessment Test Key (RAT Key)

1. Which of the following are associated with acute, episodic vomiting?
  a. Inborn errors of metabolism
  b. Food protein-induced enterocolitis syndrome (FPIES)
  c. Cannabinoid hyperemesis syndrome (CHS)
  ★. All of the above
  e. None of the above
  **Discussion:**
  Acute episodic vomiting is vomiting that presents rapidly over 24–48 hours, and occurs intermittently with periods of no vomiting. All of the above including intestinal malrotation with intermittent volvulus are associated with acute episodic vomiting (pg. 344). Chronic vomiting is typically over several days to weeks, is low volume and infrequent and rarely associated with dehydration. Chronic vomiting is associated with diseases such as peptic ulcer disease, gallbladder disease, and gastritis.
2. 1-year-old male, born full term, with no known medical problems, and up to date on immunizations, presents to the ED with his parents. They state for the past two weeks he’s been irritable and crying more than normal. They explain that he has been having “spells” where he cries and “scrunches up” or holds his stomach. They state that his stools have been getting darker, and today he had one episode of bloody stool an hour prior to arrival to the ED. On exam his abdomen is soft; however, you palpate a mass within the right lower quadrant. What is his presumed diagnosis?
  a. Pyloric stenosis
  ★. Intussusception
  c. Meckel’s diverticulum
  d. Wilm’s tumor
  **Discussion:**
  Intussusception is thought to be caused by a lead point in kids <5 (ie: Peyer’s patches from recent viral illness. In kids >5, common causes include Henoch-Schonlein purpura, lymphoma, Meckel’s diverticulum, cystic fibrosis. This child presents with intermittent, colicky generalized abdominal pain, +/− bloody stools, classically described as resembling “currant jelly.” During episodes parents describe the child as inconsolable. A physical exam may reveal a RUQ “sausage like mass.” The diagnosis is made by abdominal US, which shows a “target sign,” and treatment is an air-contrast enema, or possible surgical intervention/reduction if the enema is unsuccessful.
3. 6-week-old male, born full term via uncomplicated vaginal delivery is brought to the ED by his mother for evaluation of forceful vomiting after feeds for the past 4 days. He is up to date on immunizations. This is the mother’s first child, and she states that he seems to “always be hungry.” He is breastfed, and feeds for 30 minutes every 2 hours. Mom reports no wet diapers for the past 24 hours. You decided to get labs and they reveal a hypochloremic, hypokalemic metabolic alkalosis. What is the diagnosis?
  a. Malrotation with volvulus
  b. Breast milk jaundice
  c. Intestinal atresia
  ★. Pyloric stenosis
  **Discussion:**
  One of the most common GI causes of vomiting in infants. It is due to thickening of the pyloric muscle leading to narrowing of the pylorus. It is more common in males, age 2–7 weeks, and associated with preterm birth, drug use (such as erythromycin), small-for-gestational-age (SGA), C-Sections, and maternal smoking. Patients present with forceful, projectile vomiting. A palpable mass, “olive” in the RUQ is considered pathognomonic.
  US is the diagnostic tool of choice. Labs are usually ordered when child presents as failure to thrive and clinically dehydrated and reveal a hypochloremic, hypokalemic metabolic alkalosis. Definitive treatment is fluid resuscitation to correct electrolyte imbalance and a pyloromyotomy.
4. A 24-month-old male is brought in to the ED with by his dad for vomiting for the last 24 hours. The dad reports that the whole family has been ill since attending a lunch picnic yesterday. On physical exam the patient is mildly tachycardic, has dry mucous membranes, and a soft minimally tender abdomen. The first step in management is:
  a. Abdominal radiograph
  ★. Antiemetics and fluids
  c. Antibiotics for likely bacterial colitis
  d. NG tube for bowel decompression
  **Discussion:**
  This patient is stable, has a soft abdomen and is showing signs of dehydration. The most likely diagnosis is food poisoning. The patient can likely be given antiemetics and then give PO fluids for oral rehydration. Abdominal radiograph and/or NG (naso-gastric) tube would be appropriate if concern for obstruction or need for decompression. Antibiotics are not needed since the patient is afebrile and not sick appearing.
5. 7-month-old female, born full term and up to date on immunizations presents for evaluation of bilious emesis; parents deny bloody stools. On exam, the child is sick appearing and abdomen is distended. You order a stat KUB (kidneys, ureters, bladder) which shows air-filled levels, and dilated stomach and bowel in the upper quadrants with a paucity of air distally.
  What is your diagnosis and treatment?
  ★. Malrotation with volvulus; decompression with nasogastric intubation (NGT) and surgery consult
  f. Pyloric stenosis; pyloromyotomy
  g. Intestinal atresia; surgery consult
  h. Intussusception; air enema
  **Discussion**
  Malrotation with volvulus should always be considered in patients with acute or episodic vomiting that is bilious. During episodes of malrotation with volvulus, children may cry inconsolably, have severe abdominal pain, and be toxic-appearing. This can also occur without volvulus and lead to chronic painless vomiting.
  The diagnosis of malrotation with volvulus is usually made with abdominal x-ray (KUB) in the ER or CT abdomen/pelvis. A KUB will show an air-filled, dilated bowel proximal to the level of the obstruction and a lack of air distal to the obstruction. Treatment is decompression with NGT followed by emergent surgery consult.
  Figure 2 in “Vomiting in Children,” by T. Matthew Shields, MD, and Jenifer R. Lightdale, MD, MPH, shows an excellent example of a dilated stomach in a patient with malrotation and volvulus: https://pedsinreview.aappublications.org/content/39/7/342.
6. Which of the following are “Red Flag” signs & symptoms that require urgent evaluation?
  a. Hematemesis/Hematochezia
  b. Bilious emesis
  c. Abdominal distension/absent bowel sounds
  d. Vomiting that wakes child from sleep
  ★. All the above
  **Discussion**
  Red flag symptoms that should prompt referral for urgent evaluation by GI specialists include hematemesis (especially with the first episode of vomiting), hematochezia, recurrent bilious emesis, clinical dehydration, evidence of shock, focal neurologic changes, abdominal distention, and absent or tympanic bowel sounds. In addition, it is critical to carefully evaluate vomiting that wakes a child from sleep. These help determine the emergent causes of pediatric GI diagnosis and treatment.
7. 4-month-old female, born term and up to date on immunizations, presents to the ED for evaluation of being more irritable and fussier after feeds. She’s breastfed solely. Mother states that she has had issues with “spitting up” after feeds but has always been “happy,” and even though she appears hungry mom feels like the patient is losing weight. What is the most likely diagnosis?
  ★. GERD (gastroesophageal reflux disease)
  b. Breastfeeding jaundice
  c. Milk protein allergy
  d. Eosinophilic esophagitis
  **Discussion**
  GER is the most common physiologic causes of vomiting within infancy; it presents usually by 4 months and resolves by 1 year. Infants usually experience painless spitting up after feeds if infants are otherwise growing and developing appropriately. Infants with GER are “happy spitters,” and don’t require any acid suppressant treatment, but instead parents should be provided with reassurance. GER becomes pathologic (GERD) once there are any associated features along with GER such as weight loss, failure to thrive, poor feeding, and irritability. Treatment for GERD is conservative (smaller meals, frequent burping, sitting upright during feeds, thicken formula), Ranitidine or Reglan may be used, and if very severe, may need surgical procedure (Nissan).
8. 3-year-old boy presents with colicky abdominal pain and non-bilious vomiting. He appears non-toxic. Parents state during these episodes he cries uncontrollably and at times they wake him up from sleep for the past week. They deny bloody stools. What is the diagnostic study to best evaluate and treat this patient?
  a. Ultrasound
  ★. Air contrast enema
  c. Surgery
  d. EGD (esophagogastroduodenoscopy)
  **Discussion**
  Intussusceptions can present as a triad of abdominal pain, vomiting, and bloody stools (only presents 50% of the time). US is used to diagnose, showing a “target sign.” It presents more frequently in male patients between 3 months and 3 years of age. If air enema fails (used to diagnose and treat), the next step is surgery.
9. You are on your NICU rotation and called to come evaluate a newborn male with history of trisomy 21. The baby has been having bilious vomiting after his first feed with a distended abdomen. You order a stat KUB which shows a large gastric bubble and then a distal smaller bubble of air. What is your presumed diagnosis?
  ★. Intestinal atresia
  b. Pyloric Stenosis
  c. NEC (necrotizing enterocolitis)
  d. GERD
  **Discussion:**
  Duodenal atresia is a type of intestinal atresia that is most common in newborns, particularly with Down Syndrome, with congenital abnormalities. It presents with bilious vomiting within the first feedings of life and abdominal distention. KUB shows a classic double bubble sign—dilation of the stomach and proximal duodenum with absent distal gas. Treatment is NGT, nothing by mouth (NPO), correct any fluid/electrolyte imbalances, ampicillin and gentamicin (present post op infection), surgery.
10. An 8-year-old boy presents with acute vomiting that began after lunch yesterday with one to two episodes, but then became intractable late last night. The patient has dry mucous membranes, his abdomen is soft, non-tender and non-distended. His vital signs are HR 120, BP 90/84, RR 18. What diagnostic studies would you order?
  ★. Chem 7 & KUB
  b. Chem 7, LFTs (liver function tests), amylase/lipase, EGD, MRI/CT head
  c. Chem 7, ammonia, pyruvate, abdominal US, urine studies
  d. Urine studies only
  **Discussion:**
  Important to determine if vomiting is acute, chronic, or cyclic because this will help guide antiemetic treatment and which emetic reflex pathway to target.
  **Acute**
  - Presents rapidly 24–48hrs.
  - Associated with severe symptoms and dehydration.
  - Causes include: viral gastroenteritis, food poisoning, bowel obstruction.
  - Acute vomiting can be self-limiting or recur episodically.
  - Examples of conditions with acute, episodic vomiting: intestinal malrotation with intermittent volvulus, inborn errors of metabolism (IEMs), and food protein–induced enterocolitis syndrome (FPIES). Cannabinoid hyperemesis syndrome with severe, episodic vomiting that may be more common than previously appreciated in adolescent populations.
  **Chronic**
  - Presents with symptoms for days to weeks.
  - Rarely associated with dehydration.
  - Causes: peptic ulcer disease, gall bladder disease, eosinophilic esophagitis, gastritis, and adverse reactions to ingested foods.
  **Cyclic**
  - Intermittent episodes of sudden vomiting followed by asymptomatic periods
  - Difference between cyclic and episodic
    ○ Cyclic=stereotypical episodes & repetitive nature
      ▪ Most common etiology is cyclic vomiting syndrome (CVS), a migraine variant that involves episodes of recurrent retching and emesis leading to inability to tolerate PO and dehydration/lethargy.
  *See Shields TM, and Lightdale JR. Vomiting in Children. *Pediatrics in Review*. 2018;39(7):342–358. Table 3: Laboratory and Radiographic Evaluation for Patients with Vomiting by Temporal Pattern.
11. The pathways of emetic reflex are best described by:
  a. Motion
  b. Emotional Responses
  c. Toxins introduced by drugs/food/environment
  d. Eating too much
  ★. All the above
  **Discussion:**
  All of the above are pathways of the emetic reflex and can cause emesis.
12. 14-year-old female with history of migraines presents to the ER for evaluation of vomiting spells for the past week that occur upon waking up from sleep. She’s had an MRI and CT head that have been negative for masses. The patient’s mother states that with her migraines she only gets headaches, and never experiences nausea or vomiting. Which elements of the history are important to obtain in order to help you with your diagnosis?
  a. Social
  b. Alleviating factors
  c. Sexual & Surgical
  d. Birth
  ★. All the above
    **Discussion:**
    Cannabinoid Hyperemesis Syndrome (CHS) is associated with chronic marijuana exposure, which was first described in 2005, as a mimic of CVS. Stereotypically, CHS has hydrophilia and a history of seeking hot showers to alleviate vomiting that can help differentiate CHS from CVS. Treatment of CHS involves abstinence from marijuana for at least 2 weeks. Physicians may also use haloperidol 5mg IM and Benadryl 50mg IV/IM, Capsaicin cream 0.025–0.075% across abdomen/arms/back. Additionally, the other aspects of the history are important to rule out pregnancy, small bowel obstruction, gastroesophageal reflux disease, biliary disease, and congenital abnormalities.
  13. A 16-year-old male presents to his primary care physician’s office with complaints of episodic post-prandial vomiting. He reports that it started after he lost 20 pounds when he joined the high school football team. He reports that when he eats he gets crampy abdominal pain, but it improves if he leans forward. What do you expect to see on upper GI series?
  ★. A sharp cutoff sign where the contrast is unable to pass through the duodenum
  b. Distended small bowel with air fluid levels
  c. Diffuse constipation
  d. Dilation of the esophagus with no contrast past the lower esophageal sphincter
  **Discussion:**
  The patient’s most likely diagnosis is SMA (superior mesenteric artery) Syndrome. SMA syndrome is more common in pediatric patients that lose a significant amount of weight because the adipose tissue that normally suspends the SMA over the duodenum is reduced, causing the SMA to compresses the duodenum leading to bowel obstruction. An upper GI series will reveal a sharp cutoff sign where the contrast is unable to pass through the duodenum.

### Vomiting in Pediatric Patients TBL: Group Application Exercise (GAE) Key

#### Case 1

15-year-old female with history of Type 1 Diabetes, presents with nausea and vomiting for the past week, accompanied by her mother. Patient states last menstrual period was 2 months ago, though she has irregular periods and denies being sexually active. She denies any bloody stools, abdominal pain, and has never been in DKA per mother.

On exam, vital signs are the following: T 98.6, RR 18, HR 100, BP 110/85. The patient appears pale and dry. Abdomen is soft though slightly distended, with normal bowel sounds. Pulmonary and cardiac exam are within normal limits.

Is this patient sick, not sick, or potentially sick?

Potentially Sick. The patient has a history of Type 1 Diabetes and presents with nausea and vomiting; she is pale and dry and her abdomen is slightly distended. Her vitals are stable. However, given her history of diabetes, there is concern for DKA, and her appearing pale and dry causes immediate concern for dehydration and volume loss.

What labs/imaging/interventions/meds would you like to order?

Bloodwork—Complete blood count, complete metabolic panel, lipase
Urine—urine analysis, urine pregnancy test
Fluid bolus

You obtain the following diagnostic results listed below. At this point what is your differential diagnosis?

- WBC: 10, Hbg 12, Plt 250
- Urinalysis: no leukesterase, no WBC, no RBC, no bacteria
- CMP: within normal limits
- Blood Glucose: 100
  Gallstones, acute cholecystitis, pancreatitis, urinary tract infection, abdominal migraine, pregnancy, CHS, menstrual cycle

What other lab work would you like to order or did you order that has not resulted yet?

Urine pregnancy: Positive
Consider quantitative beta hcg

What imaging (if any) would you like to perform?

Consider a bedside US to determine intrauterine pregnancy (IUP)

What treatment would you like to provide the patient?

The patient is pale and dry; she is likely dehydrated and if tolerating po at the time could be provided oral fluids. If she is not tolerating po, which is likely given her chief concern was nausea and vomiting, then administration of intravenous fluids would be appropriate. A typical pediatric fluid bolus is 10 to 20cc/kg of normal saline.
Additionally, pregnant patients may benefit from pyridoxine (vitamin B6) given with or without doxylamine.

#### Case 2

A 24-hour-old male born via C-Section at 33 weeks secondary to mother having pre-eclampsia presents to the ED. Quad screen was positive for trisomy 21. The labor and delivery nurse calls down and states that the baby was just discharged, and prior to discharge, they noted that he had an episode of bilious emesis and progressively worsening abdominal distention. The doctor called the family and asked them to return immediately. You note a birth weight 2400g, and the baby is formula fed. No bloody stools.

On exam, VS demonstrate T 98.6, HR 145/min, BP 91/60, and RR 50. The infant is lethargic-appearing with poor skin turgor. His abdomen is distended with normal bowel sounds. Pulmonary and cardiac exam are within normal limits.

Is this patient sick, not sick or potentially sick?

Sick. This patient as noted by his lethargy and poor skin turgor. His abdominal distention should also raise concern.

What labs/imaging/interventions/meds would you like to order?

Point of care Blood Glucose, complete blood count, complete metabolic panel, urine analysis

You obtain the following diagnostic results below:

WBC: 10, Hbg 11, Plt 253
Urine: within normal limits
CMP: within normal limits

At this point what is your differential diagnosis for this patient and what do you think is the most likely diagnosis?

NEC, Intestinal/Duodenal atresia, Malrotation with volvulus, Wilm’s tumor, Hirschsprung’s disease, annular Pancreas, pyloric stenosis
Any patient with trisomy 21 who has vomiting should be evaluated for duodenal atresia. Intestinal/duodenal atresia will typically present with vomiting the first few hours of life.

You obtain the following radiograph:

Case courtesy of Dr Sebastian Tschauner, Radiopaedia.org, rID: 49592. https://radiopaedia.org/cases/49592

How would you describe the patient’s abdominal radiograph?

Abdominal radiograph with a distended stomach with a “double bubble sign,” concerning for possible obstruction such as duodenal atresia.

What is your diagnosis for this patient?

This patient most likely has duodena atresia which is a subset of intestinal atresia; the underlying cause is unknown. There are no predisposing maternal risk factors.
Presenting symptoms are typically vomiting within a few hours of birth, often bilious, although 15% of cases will be non-bilious. Vomiting is caused by an intestinal obstruction.
Intestinal atresia occurs in 1 in 2500–5000 births and 24–40% are associated with a congenital anomaly, typically trisomy 21.
Treatment is surgical, duodenoduodenostomy, typically performed within the first month of life (newborn period).
Infants may develop megaduodenum, gastritis with reflux, peptic ulcer disease.

What is the treatment for this patient?

While treatment for duodenal atresia is surgical duodenoduodenostomy and is typically performed within the first month of life (newborn period), immediate treatment should include hydration, typically with 20cc/kg normal saline, correction of any electrolyte abnormalities. Patients with significant abdominal distention may require decompression with a nasogastric tube.

What is the prognosis for patient?

Overall mortality rate can be as high as 33% (1967); however, with modern surgery it has declined to 3% in most cases. Deaths is mostly due to other associated congenital anomalies (cardiac).

#### Case 3

3-year-old male born at 36 weeks presents to the ED for evaluation of abdominal pain and being irritable for the last 3 days. Patient is fully immunized. Mother states that he has episodes of abdominal pain where he cries and grabs his stomach. After a few minutes pass, he’s “fine.” She states that he started vomiting today and has had dark red mucous colored stools as well. She looked up his symptoms on WebMD and is worried about some “vascular disease.”

On exam, vitals are T 99.6, HR 124, BP 95/63, Sp O2 97%, wt 20kg. The child is happy and playing. His mucous membranes are moist, abdomen is soft, GU exam shows an uncircumcised penis, testicles symmetric and descended, with no gross blood per rectum. Just as you are about to leave the room, the child squats down grabs his stomach and starts crying. Mother explains this is his “episode.”

Is this patient sick, not sick, or potentially sick?

Sick. This patient is having dark red mucous colored stool which should raise concern for serious pathology.

What labs/imaging/interventions/meds would you like to order?

Complete metabolic panel, complete blood count, urinalysis, ultrasound of the abdomen

You obtain the following diagnostic results below:

WBC: 12.5, Hb 14, Plt 222
Na 138, K 4.8, Cl 110, HCO 18, BUN 10, Cr 1.0, Glucose 100
AST 10, ALT 10, Alk Phos 19, Tbili 0.5, Lipase 20

Imaging shows:

Case courtesy of Dr Sally Ayesa, Radiopaedia.org, rID: 63984. https://radiopaedia.org/cases/63984

What are common causes of lower GI bleeding found in kids in this age group?

There are many common causes of lower GI bleeding in pediatrics aged 2–5:

Gastrointestinal duplication
Gastroenteritis
Upper GI bleed
Hemolytic uremic syndrome (HUS)
Henoch-Scholein Purpura (HSP)
Necrotizing Enterocolitis
Meckel’s diverticulum
Constipation with tears

What is the most likely diagnosis?

Bonus points: What are some pathological conditions associated with this condition?

The history of episodic colicky abdominal pain, with knees drawn up to chest, and “currant jelly stools” is concerning for intussusception.
The ultrasound shows a portion of the bowel (called the lead point) telescoping into the more proximal adjacent bowel.
Some pathological conditions often associated with intussusception are cystic fibrosis, HSP, Lymphoma, and Meckel’s diverticulum.

What imaging would you use to confirm and ultimately treat the patient’s condition?

Abdominal ultrasound is highly sensitive and specific and will often show a classic “bullseye.”
Barium enema confirms presences of telescoping and treats the intussusception.
The patient will also need IV hydration and electrolyte replacement.

Case courtesy of Dr Sally Ayesa, Radiopaedia.org, rID: 63984. https://radiopaedia.org/cases/63984

#### Case 4

A 16-year-old female presents for an intractable headache. She notes that the onset is first thing in the morning and that it wakes her from sleep and worsens throughout the day. Her mother states the symptoms have been progressively worsening and that the patient appears “off-balance” when she walks. Associated symptoms are nausea/vomiting; she denies blurred vision, hearing changes, weight loss, photophobia. She states her head hurts “all over.” She currently is on her menstrual period; she denies history of migraines. No pertinent family history. Social history is positive for occasional marijuana use.

On exam, vital signs are T 98.7, HR 98, RR 18, SpO2 97% RA. She is 60kg, and 5’7”. The patient is holding both sides of her head and crying; you perform a full neuro exam and notice she has drift on the right. The remainder of your exam is unremarkable.

Is this patient sick, not sick, or potential to be sick?

Sick. While the patient’s vitals are relatively normal, the patient has drift to the right upper extremity which should raise concern.

What labs/imaging/interventions/meds would you like to order?

Complete metabolic panel, complete blood count, urinalysis, urine pregnancy test, head CT, antiemetic, pain control.

You obtain the following results below:

WBC: 12, Hgb 13, Plt 235
CMP: within normal limits
UA: within normal limits
Urine pregnancy test: negative

What history elements are important to ask a pediatric patient with a headache?

Onset, duration and severity
Associated symptoms
Family history of migraines or vascular abnormalities
Medication history
Factors that may have precipitated the headache (most often migraines)
Trauma history

What is the clinical significance of the associated symptom of vomiting that the patient is experiencing?

The vomiting may be due to a generalized increased intracranial pressure or due to irritation of the vagal nuclei in medulla oblongata of the 4^th^ ventricle. It usually occurs in the morning, given to being recumbent and in hypoventilation most the night, and headache is relieved when sitting up.

What is your differential diagnosis for this patient?

Hydrocephalus
Intracranial Hypertension
Psuedotumor cerebri
Abdominal migraine
Pregnancy
Cannabinoid Hyperemesis Syndrome
Vertigo
Carotid/Vertebral dissection (if history of trauma such as football injury, neck manipulation, or riding rollercoaster)

You obtain a computed tomography of the head:

Case courtesy of Dr Hani Salam, Radiopaedia.org, rID: 10374. https://radiopaedia.org/cases/10374

What is your next step in management of this patient?

MRI, neurosurgical evaluation for posterior mass, admit to pediatrics, consider steroids.
